# Co‐occurring chronic pain and primary psychological disorders in adolescents: A scoping review

**DOI:** 10.1002/pne2.12107

**Published:** 2023-05-25

**Authors:** Sharon Bateman, Line Caes, Christopher Eccleston, Melanie Noel, Abbie Jordan

**Affiliations:** ^1^ Department of Psychology University of Bath Bath UK; ^2^ Centre for Pain Research University of Bath Bath UK; ^3^ Division of Psychology, Faculty of Natural Sciences University of Stirling Stirling UK; ^4^ Department of Psychology University of Calgary Calgary Alberta Canada

**Keywords:** adolescence, adolescents, chronic pain, co‐occurring, functioning, mental health disorder, mental health symptoms, psychological disorders, psychological symptoms

## Abstract

Long‐term health conditions, whether mental or physical, often co‐occur in adolescents. For instance, adolescents with chronic pain may experience co‐occurring primary psychological disorders. In this scoping review, we determine the influence of co‐occurring chronic pain and primary psychological disorders on adolescents' functioning. A systematic search of six databases was conducted to identify articles if they were: (1) peer‐reviewed; (2) reported original findings; (3) included participants aged 11–19 years, who experienced chronic pain (i.e., pain lasting 3 months or more) and had a co‐occurring diagnosis of a primary psychological disorder; and (4) assessed functioning. Searches returned 9864 articles after the removal of duplicates. A two‐phase abstract and full‐text screening process identified two eligible articles which compared emotional functioning (*n* = 1) and social functioning (*n* = 2) between groups of adolescents with co‐occurring chronic pain and primary psychological disorders with adolescents only reporting chronic pain. Overall findings revealed no differences in social functioning, but adolescents with co‐occurring chronic pain and a primary psychological disorder (depression and anxiety) reported worse emotional functioning compared with adolescents with chronic pain alone. This review confirms the limited research on the co‐occurrence of primary psychological disorders and chronic pain in adolescents by only identifying two eligible articles exploring the co‐occurrence of chronic pain with depression, anxiety, and/or attentional disorders.

## 1 INTRODUCTION

As a developmental time‐point, adolescence comprises substantive changes in biological, social, and psychological functioning.^1^, ^2^ The period of adolescence is typically considered to span the ages of 10–19 years,^3^ although recent calls have been made to extend the period of adolescence from 10–24 years.^2^, ^4^ Beginning with the onset of puberty, the transition from childhood to adulthood involves physical, cognitive, social, emotional, and behavioral transformations, which some young people experience as challenging or distressing.^5^, ^6^ These normative developmental transformations are often accompanied by adolescents' experience of psychological symptoms.^7^ Indeed, many psychological disorders begin in childhood or adolescence and increase with age.^7^, ^8^ The World Health Organization (WHO)^3^ estimates the incidence of primary (diagnosed DSM‐IV disorders) psychological disorders in adolescents to range from 10 to 20%, with depression and anxiety being the most commonly reported.

Living with primary psychological disorders is associated with a wide‐ranging and deleterious impact on the lives of some adolescents, with a substantial number reporting untreated psychological disorders persisting into adulthood.^9^ Approximately 50% of adolescents who experience psychological disorders will experience a recurrence of their initial disorder, or develop a different psychological disorder in adulthood.^10^ Additionally, adolescents who experience elevated psychological symptoms often develop co‐occurring psychological and physical health conditions. For example, adolescents who experience insomnia are also more likely to develop disorders such as depression, anxiety, and chronic pain in adolescence.^11^ Accumulating evidence emphasizes the co‐occurrence of chronic pain (pain that occurs for 3 months or longer^12^) and psychological disorders. For example, depression often co‐occurs with chronic pain in children and adolescents.^13^ The co‐occurrence of chronic pain and psychological disorders was also found in a large nationally representative study of adolescents who experienced co‐occurring chronic pain and diagnosed anxiety.^14^ Interestingly, the above study found that adolescents were more likely to experience psychological disorders *before* the onset of chronic pain rather than following the onset of chronic pain.

Prevalence rates of chronic pain vary across studies, depending on the methods used. A systematic review identified rates of 11–38% of young people reporting an episode of recurrent or persistent pain^15^ with elevated rates of pain reported in girls compared with boys. Adolescents with chronic pain often experience challenges to functioning, such as impaired developmental,^16^ physical,^17^ and social functioning.^18^ For many young people, the experience of chronic pain is also associated with co‐occurring psychological symptoms, with adolescents who experience chronic pain being twice as likely to report high levels of emotional distress when compared to adolescents without chronic pain.^19^ As such, a number of adolescents living with chronic pain may subsequently develop a primary psychological disorder. This is supported by evidence from Shelby et al.^20^ who found that in a sample of adolescents (8–17 years old at baseline) with functional abdominal pain, 30% met diagnostic criteria for an anxiety disorder 4 years later, with heightened risk for the development of a primary psychological disorder continuing even when the chronic pain had resolved. Conversely, evidence of bi‐directional relationships reveals that some children and adolescents who experience poor psychological symptoms subsequently develop chronic pain in adulthood.^21^, ^22^ These co‐occurring chronic pain and psychological symptoms often negatively impact adolescent functioning.

With regard to functioning, adolescents with co‐occurring chronic pain who report higher levels of psychological symptoms (e.g., depressive symptoms) often experience more impaired social functioning, functional disability, and increased school absenteeism than adolescents without elevated depressive symptoms.^23^ Furthermore, for adolescents who experience chronic pain, the addition of depression and anxiety symptoms are consistently associated with greater levels of disability.^24^ Importantly, research has typically focused on adolescents with co‐occurring chronic pain and psychological symptoms whether clinically diagnosed or not. Despite the frequency of co‐occurring chronic pain and psychological disorders,^14^, ^25^ little is known about the levels of functioning of adolescents with co‐occurring chronic pain and primary psychological disorders. In this scoping review, we aim to address this gap by identifying and summarizing the current evidence regarding the impact of co‐occurring chronic pain and primary psychological disorders on adolescent functioning. Additionally, we will use findings to outline a research strategy on how to advance this line of inquiry. We will focus on studies that report on adolescents aged 11–19 (to capture those of secondary school age in the English education system), who experience both chronic pain and primary psychological disorders, with a focus on the effects of co‐occurring presentation on functioning. Only articles that include explicit observations of adolescents with both chronic pain and primary psychological disorders (those fulfilling DSM‐5 criteria) will be included in our review.

## 2 METHODS

A scoping review^26^ was chosen to identify emerging evidence that informs practice in the field as well as knowledge gaps. Our review, guided by methodological frameworks authored by Arksey and O'Malley^27^ and Peters et al.^28^ was conducted to map and synthesize the literature regarding the functioning of adolescents with co‐occurring chronic pain and psychological symptoms. The scoping review protocol is available on the Open Science Framework at https://osf.io/sjvq2/. The Arksey and O'Malley^27^ methodological framework comprises five stages: (1) identification of a research question; (2) identification of relevant studies; (3) study selection; (4) charting the data; (5) collating, summarizing, and reporting the results. Additionally, and outside of the stages recommended by Arksey and O'Malley, the quality of the included articles was assessed using the “scientific merit appraisal method” developed by Alderfer et al.^29^ (see Appendix S1) for full criteria and results. However, this assessment was not used in consideration of inclusion or exclusion of the articles in the review and was merely a tool for the authors to evaluate the included papers.

### 2.1 Stage 1: Identifying the research question

We asked: What is known about the functioning of adolescents with co‐occurring chronic pain and primary psychological disorders?

### 2.2 Stage 2: Identifying relevant studies

The five key concepts to be covered by the search strategy were identified by the first author (S.B.) during a broad literature search. These concepts comprise: (1) *adolescence*; (2) *co‐occurrence*; (3) *primary psychological disorder*; (4) *chronic pain*; (5) *patterns of functioning*. The electronic literature search was conducted across six databases, Embase, PsycNET, PubMed, Web of Science (Core Collection), PsycINFO, and CINAHL with the last search conducted on 17th February 2020. To ensure that all five concepts were captured, two search strings were developed, string one: age, primary psychological disorder, chronic pain, and co‐occurrence and string two: age, primary psychological disorder, chronic pain, and function. Search terms for each string were selected to cover the relevant concepts. Table 1 shows examples of search terms for each concept, the full list can be seen in Appendix S2.

**TABLE 1:** Search terms used to conduct a literature search.

Age	Adolesc* OR Child OR Children OR “Late childhood” OR “Early adulthood”
Primary psychological disorder	“Autism Spectrum Disorder” OR ASD OR * OR OCD OR PTSD
Chronic pain	“Chronic Pain” OR Pain OR “Long term pain” OR “Persistent pain”
Co‐occurrence	Co occur* OR Comorbid* OR Co morbid* OR “Dual Diagnosis”
Function	Function* OR Academi* OR School OR Social* OR Emotion*

*Note*: This table demonstrates an example of how the search terms for each string were used and is not a comprehensive presentation of the search terms used.

**FIGURE 1:**
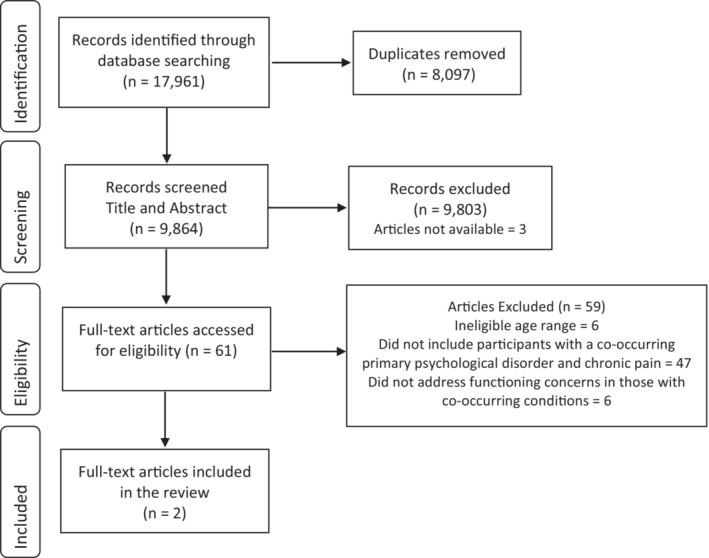
PRISMA flow chart of scoping review search.

Searches were conducted in each database by first searching using string one followed by a separate search for string two. For each database, both searches were limited to the title and abstract fields, peer‐reviewed articles, original studies, and articles written in the English language. No publication date was specified. A total of 17 961 references were retrieved and uploaded to Endnote X9^30^ and subsequently, to Covidence software (www.Covidence.org). Duplicates were removed, (*n* = 8097), with the remaining 9864 references retained for the title and abstract screening, see Figure 1 for the PRISMA diagram of the screening process.

### 2.3 Stage 3: Study selection

Covidence was used to screen the final selection of 9864 articles following a two‐step approach.

### 2.4 Step One: Screening; abstract and title search

The screening was completed on the 9864 selected articles by the first author (S.B.). A total of 25% of the eligible articles at this stage were double‐screened by independently trained research assistants. Title and abstract screening were conducted using clear and well‐defined inclusion and exclusion criteria (See Table 2 for inclusion and exclusion criteria). Articles were excluded at this stage if they: (1) included participants outside of the age range of 11–19 years; (2) did not provide a measure of functioning of adolescents who experience co‐occurring chronic pain and primary psychological disorder; (3) were not peer‐reviewed, or (4) did not report original empirical findings. A small number of conflicts *n* = 4, where a consensus could not be reached, were retained for full‐text screening. Despite an exhaustive search, the inter‐library loans service and contacting authors, a further *n* = 3 articles (see Appendix S3) had to be excluded as a full text could not be sourced. In line with guidance for systematic reviews from Siddaway et al.^31^ the reasons for rejection were not retained at this stage; however, the number of rejected articles was noted (i.e., *n* = 9803), with the remaining *n* = 61 articles progressing to the full‐text review.

**TABLE 2:** Study inclusion criteria.

Peer‐reviewed articles.
Reported on original findings.
Full‐text articles were written in English.
Included participants aged 11–19 years where separate data is reported for any age group within the age range 11–19.
Included participants who experienced diagnosed primary psychological disorder and chronic pain, that lasted 3 months or more, including self‐report and parent‐report studies.
Addressed concerns around functioning in adolescents who experience diagnosed primary psychological disorder and experience chronic pain (pain lasting 3 months or more that was continuous or intermittent).

### 2.5 Step Two: Screening; full‐text review

A full‐text review of eligible articles (*n* = 61) was conducted by the first author S.B., and trained research assistants. All articles were double screened with a further 50% screened, for added rigor, by the research assistant not involved in the initial double screening of the article. Conflicts were resolved by A.J. A total of *n* = 2 studies were found to be eligible for inclusion at this stage following confirmation of the inclusion criteria (see Table 2). Fifty‐nine articles were eliminated with the majority not meeting the criteria for participant eligibility of diagnosed primary psychological disorder and chronic pain (*n* = 47). Following confirmation of the eligible articles, a thorough backward citation search was conducted on each paper to ensure that eligible articles were not overlooked.

### 2.6 Stage 4: Charting the data

A data extraction form was devised by the first author S.B. (Appendix S4) with data charted to show the following: (1) article details, for example, author, year and location of publication, study aims and objectives; (2) participant information e.g., sample size, age range, gender and culture, pain and /or psychological disorder; (3) study design, i.e., longitudinal, cross‐sectional, quantitative or qualitative; (4) the analytical approach/methods used; (5) methodology e.g., the assessment type; (6) outcomes or key findings; (7) interpretation of outcomes; and (8) any other relevant information.

### 2.7 Quality assessment

The quality of the included articles was assessed using the ‘scientific merit appraisal method’ developed by Alderfer et al.^29^ to allow for a rigorous assessment of published articles irrespective of their methodology (see Appendix S1 for full criteria and results) and frequently used to record the quality of published papers in reviews.^32^, ^33^ Although a quality assessment is outside of the Arksey and O'Malley^27^ methodological framework used for this scoping review, the appraisal of quality provided a robust method to allow the direct comparison across articles. Articles were ranked by individual grading between 1 (low quality) and 3 (high quality) on rating criteria of 9, 11, and 16, respectively. The quality assessment was independently conducted by S.B. and trained research assistants. Articles were not excluded as a result of their quality assessment score and there were no disagreements regarding the quality of the included articles recorded.

## 3 RESULTS

### 3.1 Stage 5: Collating, summarizing, and reporting the results

Two articles were included in this review and comprised participants from Taiwan, Wang et al.^34^ and the USA, Kashikar‐Zuck et al.^35^ Across the articles, the number of participants with co‐occurring pain and primary psychological disorders was recorded as *n* = 121, recruited from a community sample, mean age 13.9 years and were predominantly female 74.4% (Wang et al.) and *n* = 102, recruited from a specialist pain center, mean age 14.96 and were also predominantly female 87.3% (Kashikar‐Zuck et al.). Both articles adopted a quantitative approach and incorporated a cross‐sectional design using self‐report assessment surveys and an additional diagnostic psychiatric interview, following the Diagnostic and Statistical Manual of Mental Disorders (4th ed.; DSM‐IV^36^). A descriptive summary of the included articles can be seen in Table 3.

**TABLE 3:** Descriptive Summary of Included Articles.

Article attributes	Kashikar‐Zuck et al., 2010	Wang et al., 2007
Sample		
Whole sample size	102	121
Group of interest sample size	102	57
Age range of article	11–18	12–15
Mean age	15	13.8
% Females in whole sample	(87.3%)	(74.4%)
% Females with co‐occurring pain and mental health symptoms	(87.3%)	52%
Recruited from		
Pain clinic	x	
Community		x
Location		
USA	x	
Taiwan		x
Methods		
Quantitative	x	x
Design		
Cross‐sectional	x	x
Assessment methods		
Self‐report	x	x
Clinical evaluations	x	x
Parent report	x	
School report	x	
Pain conditions		
Chronic daily headache		x
Fibromyalgia	x	
Mental health conditions		
Anxiety		x
Depression	x	x
Attentional disorders	x	
Functioning domain assessed		
Social	x	x
Emotional		x

With respect to the overall research question, of identifying the nature of adolescent functioning, Wang et al.^34^ focussed on examining the prevalence of primary psychological disorders, in participants with a chronic pain condition, to explore the effects on the varied patterns of functioning. In contrast, Kashikar‐Zuck et al.^35^ focussed on exploring the relationships between chronic pain and functional difficulties, such as school absenteeism. Both articles focused on studying specific types of pain conditions, Juvenile Idiopathic Arthritis (JIA) in the case of the Kashikar‐Zuck et al.^35^ article and chronic daily headache in the Wang et al.^34^ paper (see Table 3 for full overview).

### 3.2 Assessment of co‐occurrence

Regarding measurement, Kashikar‐Zuck et al.^35^ used the Children's Depression Inventory (CDI),^37^ to assess adolescent self‐report of depression and the Kiddie Schedule for Affective Disorders and Schizophrenia (K‐SADS‐PL)^38^, ^39^ to conduct the diagnostic semi‐structured psychiatric interview. The CDI has been validated for the age group 7–17 years, and the K‐SADS‐PL is a validated measure for the age group 7–17 years. In addition to the adolescent measures, parents provided a report of adolescent pain history, and school attendance was recorded via both parent report and school report in the Kashikar‐Zuck et al. article. Additionally, adolescents in this study reported their average pain duration as 35.77 months, and although the self‐report depressive symptoms on the CDI were mildly elevated in comparison to age norms, 25% of the participants reported scores above the clinical cut‐off (T‐score = 65) for depressive symptoms. Furthermore, 24% of adolescents met the criteria for an attentional disorder, and 19% met the criteria for a depressive disorder. In comparison, in the Wang et al.^34^ paper, adolescents with a chronic daily headache diagnosis (experienced headaches for 2 hours or more per day, for 15 or more days per month) participated in diagnostic psychiatric interviews using the Structured Mini‐International Neuropsychiatric Interview‐Kid for children and adolescents (MINI‐Kid),^40^ a validated measure for the age group 6–17 years. Furthermore, 67% of adolescents in this study reported symptoms of migraine or probable migraine, 55% experienced migraine without aura and 12% experienced migraine with and without aura. With regard to psychological symptoms, 47% had at least one depressive or anxiety disorder; the most frequent were major depression 21% and panic disorder 19%. The subsequent narrative part of the review will summarize the evidence on the identified functioning domains of emotional and social functioning.

### 3.3 Domains of functioning

#### 3.3.1 Social functioning

Social functioning was examined in both the Kashikar‐Zuck et al.^35^ and the Wang et al.^34^ articles.

Social functioning was operationalized as school attendance in the Kashikar‐Zuck et al.^35^ article and the number of sick days (referred to as sick leave) taken in the Wang et al.^34^ Although neither article included in this domain specified the need for a diagnosed psychological disorder in their inclusion criteria, both articles sought to examine the prevalence of co‐occurring chronic pain and clinical levels of psychological disorders and/or the impact of having co‐occurring symptoms on social functioning. However, only Kashikar‐Zuck et al.^35^ included the explicit objective to assess social functioning in participants experiencing chronic pain and a psychological disorder, while Wang et al.^34^ set out to investigate the prevalence and association of psychological disorders in adolescents with chronic pain.

Kashikar‐Zuck et al.^35^ concluded that there were three separate primary psychological disorders prevalent in adolescents with chronic pain, notably depression, anxiety, and attentional disorders. The authors also identified an association between social functioning and school absenteeism. However, they concluded that for adolescents with chronic pain, the diagnosis of a primary psychological disorder did not significantly increase the number of school days absent when compared to those without a primary psychological disorder.

Wang et al.^34^ reported the existence of two separate primary psychological disorders in their adolescent sample, depression, and anxiety. Results showed no difference in social functioning when comparing adolescents experiencing co‐occurring pain and primary psychological disorders with adolescents who experience chronic pain alone.

To conclude, both articles investigated the domain of social functioning and reported no significant differences between adolescents who experienced chronic pain and co‐occurring primary psychological disorders and those adolescents who experienced chronic pain alone.

#### 3.3.2 Emotional functioning

Whilst the evaluation of emotional functioning was not the primary aim of the Wang et al.^34^ article, emotional functioning was examined as an adjunct to their primary objective. Wang et al.^34^ identified suicide risk in participants with chronic daily headache (CDH), which we have operationalized as emotional functioning. In their study, Wang and colleagues reported that female participants who experienced CDH and additional co‐occurring primary psychological disorders (i.e., major depression and anxiety disorder) reported elevated levels of suicide risk as compared to participants with CDH alone.

## 4 DISCUSSION

In this review, we set out to identify and summarize the current evidence and any gaps in the literature regarding the impact of co‐occurring chronic pain and primary psychological disorders on adolescent functioning. We identified only two articles with a focus on co‐occurring chronic pain and primary psychological disorders, with an emphasis on functioning, highlighting a dearth in the literature. Specifically, we only included articles that reported independent results for adolescents in our selected age range (11–19 years) who experienced chronic pain and were also diagnosed with primary psychological disorders by diagnostic clinical interview. Both eligible studies adopted a cross‐sectional approach to studying the co‐occurrence of chronic pain and primary psychological disorders in adolescents. Regarding our objective to examine functioning, we found that one of the two eligible articles revealed that adolescents with co‐occurring chronic pain and primary psychological disorders reported poor emotional functioning, which was also worse for these adolescents when compared to adolescents with chronic pain alone. This finding is congruent with previous research that reported more impaired emotional functioning among adolescents who experience co‐occurring chronic pain and psychological symptoms.^41^ However, it is important to note that Paschoal and colleagues^41^ did not consider diagnosed primary psychological disorders specifically. Regarding social functioning, our findings reveal that neither eligible article found significant differences in social functioning between adolescents who experience chronic pain and primary psychological disorders and adolescents' pain alone. This finding is in contrast to articles exploring the impact of high levels of psychological symptoms (e.g., depressive symptoms) in adolescents with chronic pain (e.g., Kasikar‐Zuck et al.^35^; Al‐Khotani et al.^42^; Gauntlett‐Gilbert et al.^43^; Gauntlett‐Gilbert & Eccleston,^23^ Paschoal et al.^41^) who show worse social functioning than their peers with chronic pain alone.

Perhaps the most critical finding in our review concerns the lack of research surrounding the co‐occurrence of chronic pain and primary psychological disorders, meaning that little is known regarding the functioning of adolescents who experience both chronic pain and primary psychological disorders. Existing literature typically focuses on assessing the functioning of adolescents recruited from chronic pain clinics who report chronic pain and elevated levels of psychological symptoms via self‐report measures.^43^, ^44^ Similarly, previous literature also reveals that adolescents recruited through mental health settings also report chronic pain symptoms^45^; however, there was no reference to functioning outside of pain‐related disability in this article. While research concerning elevated psychological symptoms helps provide some context, there are important differences between adolescents who experience symptom elevations and those who are diagnosed, meaning that adolescents who present in mental health settings with diagnosable psychological conditions and accompanying chronic pain are often not represented or identifiable in the literature. Chronic pain does not inoculate an individual against a primary psychological disorder, so we would expect up to 20% of adolescents with chronic pain to also have a history of, or a current, primary psychological disorder. Currently, it is not possible to identify this subpopulation and would undoubtedly make any investigations of sub‐groups and approaches to treatment or assessment of differential outcomes impossible.

Interestingly, during the screening process, we observed a variation in how the presence of psychological disorders was assessed. Our review only included articles that provided a diagnosis of primary psychological disorders, meaning that articles that considered elevated symptoms on a range of psychological assessment measures were excluded. Adhering to such a strict criterion provided a focused overview of the evidence of co‐occurrence of diagnosed conditions yet resulted in the exclusion of research focussed on co‐occurring symptoms. However, the exclusion of articles that discuss high or significantly high scores or scores above clinical cut‐offs of psychopathology (*n* = 9) reveals that research surrounding the co‐occurrence of chronic pain and primary psychological disorders in an adolescent population is being conducted, but the methods used to assess psychopathology are too varied and require reconsideration. Future research is needed to review how psychological measures are used in an adolescent chronic pain population and to formulate a strategy whereby we can ensure consistency and rigor when discussing diagnosed disorders and not elevated symptoms. Whilst we acknowledge that clinical interviews may produce more conservative estimates versus symptom measures, and might be onerous on research resources, the addition of clinical interviews to diagnose psychological disorders would be a valuable addition. For example, a recent study by Vehling et al.^46^ investigated cancer among a large well‐phenotyped sample of adults with age‐matched disease‐free controls. Participants were screened for psychological disorders and further assessed with a diagnostic clinical interview. The authors were then able to examine clinically relevant comorbidities, in this case, affective disorders, which revealed interesting results in that cancer may be associated with both increased and decreased prevalence rates for psychological disorders. The Vehling et al. study^46^ highlights that it is indeed possible to successfully conduct high‐quality, well‐defined studies with clear, rigorous assessment when assessing clinically relevant comorbidities.

Variety in methods used to assess primary psychological disorders also showcases how research surrounding psychopathology in a chronic pain population has understandably developed to foreground pain‐specific rather than general psychology, particularly since evidence highlights how adolescents who are not typically anxious report anxiety related to their pain.^47^ However, when we consider that the wider population of adolescents who experience at least one psychological disorder is currently 10–20%.^3^ Then, we assume that this figure would be consistent or even higher for those adolescents who also experience chronic pain. Pain‐related psychological functioning is, of course, important to consider, but perhaps in considering it so well, we are now occluding primary psychological disorders in the chronic pain population.

Furthermore, given our findings identified only depression and anxiety as primary psychological disorders to co‐occur with adolescent chronic pain and negatively impact functioning, further research is needed to explore a wider range of psychological disorders. Examples include autism, eating disorders, attention deficit hyperactivity disorder, and bipolar disorder. Moreover, focusing on Posttraumatic stress disorder (PTSD), evidence shows that in comparison to young people without chronic pain, those with chronic pain reported significantly higher PTSD symptoms, and those symptoms were clinically elevated and related to worse pain and functioning.^48^ We suggest that by expanding the investigations to examine a wider range of psychological disorders, a more thorough understanding of the functioning experienced by adolescents who live with co‐occurring chronic pain and primary psychological disorders can be generated. At this point, we can only speculate as to the lack of research evidence regarding other co‐occurring primary psychological disorders. One potential reason could be the high prevalence of anxiety and depression that is often seen in the non‐pain adolescent population. Thus, the understanding of the co‐occurrence of these disorders is more readily embraced by researchers. Second, to avoid participant burden due to lengthy questionnaire batteries, researchers may use more familiar, frequently used measures to investigate known psychological disorders rather than more time‐intensive yet rigorous measures to assess for diagnosable psychological conditions (e.g., diagnostic clinical interviews that require training to administer). Regardless of the reason for this lack of variety in assessment measures, we recommend using assessment measures with clinical cut‐offs and or those with screening tools to enable branching, allowing those adolescents that screen high to be subsequently assessed for disorders, for instance, with diagnostic clinical interviews such as the Kiddie‐SADS.^39^ We advocate for future exploration to determine the prevalence of primary psychological disorders in the adolescent chronic pain population and the surrounding challenges to functioning faced by adolescents who experience co‐occurring chronic pain and primary psychological disorders compared to those experiencing a single symptom. Until we gain greater insight into these co‐occurring conditions and patterns of functioning in adolescents, we will continue to provide the closest matched therapies and treatments to adolescents, which may or may not help in their unique situation. Work that could inform tailored treatments for other co‐occurring conditions shows how unified treatment protocols have been used to treat co‐occurring psychological disorders^49^ and have the potential to treat co‐occurring conditions, such as chronic pain and primary psychological disorders. Furthermore, transdiagnostic approaches that target common mechanisms across co‐occurring conditions have previously been shown to improve co‐occurring chronic pain, anxiety, and depression symptoms in several case studies and warrant further development to explore whether similar findings can be found in a wider variety of primary psychological disorders.^50^, ^51^ With respect to chronic pain, existing mutual maintenance models (e.g., depression,^13^ and PTSD^52^) can be used to identify common underlying mechanisms and treatment targets (e.g., sleep disturbances).

Reflective of wider chronic pain and psychological research literature,^9^, ^53^ participants with co‐occurring symptoms included in the articles within this review were predominantly female. This dominance is often observed in chronic pain and psychological research literature. Beyond this female‐dominant literature, evidence also mainly stems from Western countries with a sample identifying as predominantly white. Consequently, the generalization of the findings might be limited, and further research is needed in more diverse samples. However, in this instance, the studies were conducted in the USA and Taiwan and did offer some diversity. Furthermore, whilst this review included a comprehensive search strategy, we recognize the difficulty in developing an entirely comprehensive search criterion and acknowledge that this could be a possible limitation of our review. Finally, regarding the design of future studies, we recommend that chronic pain and psychological research be focussed on longitudinal exploration. The lack of longitudinal research may result in missing key information surrounding the adolescent experience over time and which interventions or treatments are successful over time. In addition, future research should ensure that adolescents are recruited from both chronic pain and mental health clinics to ensure that we garner a greater understanding of any differences between the primary conditions.

## 5 CONCLUSION

In conclusion, the current literature on co‐occurring chronic pain and primary psychological disorders in adolescence is limited, despite the increasing numbers of adolescents reporting these co‐occurring symptoms. Our review particularly highlights how the limited research being conducted on chronic pain and primary psychological disorders is not focussing on a clinical diagnosis for primary psychological disorders, and instead, most of the time relies on elevated scores on symptom measures. This is problematic, as we know adolescents are experiencing these symptoms at a clinical level, and failure to acknowledge these disorders may mean that they go untreated. Furthermore, although this review includes two articles, it is clear from our review that adolescents with these co‐occurring conditions face more challenges to emotional functioning in comparison to adolescents who experience chronic pain alone. It is also clear that the psychological disorders and the assessment measures used to investigate them are often limited to depression and anxiety, with little exploration of the different types of psychopathologies that many adolescents experience. Subsequently, research surrounding co‐occurring chronic pain and psychopathology needs to be expanded to include a rigorous assessment of common adolescent psychopathology, including self‐harm, conduct disorder, eating disorders, OCD, ADHD, PTSD, phobias, etc.

## CONFLICT OF INTEREST STATEMENT

This work was undertaken by the named authors of the manuscript and was funded by the Pain Relief Foundation and the Sir Halley Stewart Trust. The views expressed within this report are those of the authors and not necessarily those of the Foundation or the Trust.

I can confirm that all authors have contributed substantially to the manuscript and have reviewed and agreed to the content. The authors report no conflicts of interest.

## Supporting information


Appendix S1.

Appendix S2.

Appendix S3.

Appendix S4.

## Data Availability

The data that supports the findings of this study are available in the supplementary material of this article.
