# Supplementary material for: Co‐occurring chronic pain and primary psychological disorders in adolescents: A scoping review
Source: Paediatr Neonatal Pain. 2023 May 25;5(3):57–65. doi: 10.1002/pne2.12107 (PMC10514777; doi:10.1002/pne2.12107)
Supplement: Supplementary file 1 — Appendix S1. [file PNE2-5-57-s003.docx]

Supplementary Material 1

Quality assessment Tool, Full Criteria

| **All Studies**   1. Explicit scientific context and purpose: Clear rationale for the study, theoretically and/or empirically based questions/predictions, specified purpose. 2. Methods: design and analysis are appropriate to the question(s) posed; methods are described adequately, allowing for replication of the procedure. |
| --- |
| **Quantitative Studies**   1. Measurement reliability and statistics: variables are measured reliably; ﻿proper (adequate, even if not best) statistical methods are used ﻿statistical results are appropriately interpreted. 2. Statistical power is sufficient. 3. Internal validity ﻿If two groups are compared, groups are comparable in ﻿all aspects except the IV or appropriate steps are taken ﻿to equate participant characteristics. ﻿If groups are examined over time, only the passage of ﻿time occurred between assessments ﻿If correlations (e.g., X-Y) are examined, the reverse ﻿relationship is not feasible, and a third variable could not be ﻿causing both X and Y. 4. Measurement validity and generalisability of constructs: variables ﻿are appropriately operationalized and measured; results would be ﻿the same if other measures were used. 5. External validity: The findings are generalizable to the target ﻿population, real world, and across time periods. |
| **Qualitative and mixed Methods Studies**   1. ﻿Grounding in examples: examples illustrate conclusions, help reader ﻿understand analytic procedure and form possible alternative meanings statistical ﻿of the data. 2. Findings are integrated into a framework. 3. ﻿Owning one’s perspective: Authors specify their theoretical orientation ﻿and expectations that might impact interpretation of data. 4. ﻿Resonating with readers: Accurate, understandable perspective of ﻿topic area. 5. ﻿Data is based on an appropriate range of informants or situations and/or ﻿the topic has been studied systematically and comprehensively within the ﻿specified situation or population. 6. ﻿Credibility checks: verification of findings with participants, across ﻿multiple coders, or through methodological triangulation. 7. ﻿Situating the sample: Sample is described such that the reader can ﻿judge for whom the findings are relevant. |
| **All Studies continued**   1. **﻿**Appropriate discussion: limitations noted; conclusion appropriate to data gathered. 2. ﻿Contribution to knowledge: contributing something new or validating past results. |
| **Totals** |
| 1. Total scores for studies 2. Average of scores divided by 9 for quantitative studies. Divided by 11 for qualitative studies and 16 for mixed methods studies |

*Note.* Alderfer et al., (2010) criteria for quality assessment tool used to assess the quality of articles included in the scoping review. Letters equate to columns in table.

Supplementary Material 1

Quality Assessment Table

|  |  | **All studies** | | | **Quantitative studies** | | | | | **Qualitative Studies** | | | | | | | **All Studies** | | **Total scores** | | **Average scores** | |
| --- | --- | --- | --- | --- | --- | --- | --- | --- | --- | --- | --- | --- | --- | --- | --- | --- | --- | --- | --- | --- | --- | --- |
| **Reviewer** | **Paper code** | **﻿A** | | **B** | **﻿C** | **﻿D** | **E** | **F** | **﻿G** | **﻿H** | **﻿I** | **J** | **﻿K** | **﻿L** | **﻿M** | **N** | **O** | **P** | | **Q** | | **R** |
| SB | 1 | | 2 | 3 | 3 | 2 | 3 | 3 | 2 |  |  |  |  |  |  |  | 2 | 2 | | 22 | | 2.4 |
| AD | 1 | | 2 | 3 | 3 | 2 | 3 | 3 | 2 |  |  |  |  |  |  |  | 2 | 2 | | 22 | | 2.4 |
| SB | 2 | | 3 | 3 | 3 | 1 | 3 | 3 | 2 |  |  |  |  |  |  |  | 3 | 3 | | 24 | | 2.7 |
| AD | 2 | | 3 | 3 | 3 | 1 | 3 | 3 | 2 |  |  |  |  |  |  |  | 3 | 3 | | 24 | | 2.7 |
| *Note.* Article code 1 denotes authors, Wang, S-J, Juang, K-D, Fuh, J-L and Lu, S-R. (2007). Neurology, 68 (18), 1468–1473. Article code 2 denotes authors, ﻿Kashikar-Zuck, S., Johnston, M., Ting, T.,Graham, B., Lynch-Jordan, A., Verkamp, E., Passo, M., Schikler, K., Hashkes, P., Spalding, S., Banez, G., Richards, M., Powers, S., Arnold, L. & Lovell, D. (2010). Articles rated 1-3 in each category; 1 = poor quality, 3 = high quality, total scores possible are divided by 9. | | | | | | | | | | | | | | | | | | | | | | |
