# Supplementary material for: Co‐occurring chronic pain and primary psychological disorders in adolescents: A scoping review
Source: Paediatr Neonatal Pain. 2023 May 25;5(3):57–65. doi: 10.1002/pne2.12107 (PMC10514777; doi:10.1002/pne2.12107)
Supplement: Supplementary file 2 — Appendix S2. [file PNE2-5-57-s004.docx]

Supplementary Material 2

| Supplementary Table 2  *Full List of the Search Terms Used to Conduct the Literature Search to Identify Eligible Articles Across Databases* | |
| --- | --- |
| Concept covered | Search term used in strings |
| Age | Adolesc* OR Child OR Children OR “Late childhood” OR “Early adulthood” OR “Young Adult” OR Youth* OR “Young People” OR “Young person” OR Juvenil* OR Paediatric* OR Pediatric* OR Youngster OR Teen* OR Minor* Or Pubesc* |
| Primary psychological disorder | Neurodevelopmental-Disorder* OR “Neuro Developmental Disorder” OR “Neuro Developmental Disorders” OR Intellectual Disab* OR “Intellectual Developmental Disorder” OR “Intellectual Developmental Disorders” OR “Global Developmental Delay” OR “Unspecified Intellectual Disability” OR “Unspecified Intellectual Disabilities” OR Communication-disorder* OR Language-Disorder* OR “Speech Sound Disorder” OR “Speech Sound Disorders” OR “Childhood Onset Fluency Disorder” OR “Childhood Onset Fluency Disorders” OR Stutter* OR “Social Pragmatic Communication Disorder” OR “Social Pragmatic Communication Disorders” OR “Unspecified Communication Disorder” OR “Unspecified Communication Disorders” OR “Autism Spectrum Disorder” OR ASD OR “Attention Deficit Hyperactivity Disorder” OR “Specific Learning Disorder” OR “Specific Learning Disorders” OR Motor-Disorder* OR “Stereotypic Movement Disorder” OR “Tic Disorder” OR “Tourette Disorder” OR Schizo* OR Psycho* OR Delusional* OR Catatoni* OR Bipolar* OR Hypomanic OR Manic OR Cyclothymic* OR Depress* OR Dysthymi* OR Dysphoric* OR Anxi* OR “Selective Mutism” OR Phobi* OR “Obsessive Compulsive Disorder” OR “Obsessive Compulsive Disorders” OR OCD OR Compulsi* OR Dysmorphi* OR Hoarding-Disorder* OR Trichotillomania OR “Hair Pulling Disorder” OR “Excoriation Disorder” OR “Skin Picking” OR Attachment-Disorder* OR “Disinhibited Social Engagement Disorder” OR Posttrauma* OR Post-trauma* OR PTSD OR “Acute Stress Disorder” OR “Acute Stress Disorders” OR Adjustment* OR “Trauma and Stressor Related Disorder” OR “Trauma and Stressor Related Disorders” OR Dissociative* OR "Depersonalization Derealization Disorder” OR Somatoform* OR Somatic* OR Somatization OR “Illness Anxiety Disorder” OR “Illness Anxiety Disorders” “Conversion Disorder” OR “Functional Neurological Symptom Disorder” OR Factitious* OR “Feeding or Eating Disorder” OR “Feeding or Eating Disorders” Pica* OR Rumination* OR “Avoidant Restrictive Food Intake Disorder” OR “Avoidant Restrictive Food Intake Disorders” OR Eating-Disorder* OR Anorexia* OR Restrict* OR Binge* OR Purg* OR Bulimia* OR “Elimination Disorder” OR Enuresis OR Encopresis OR Insomn* OR Hypersomnolen* OR Hyper-somnolen* OR Narcolep* OR “Breathing Related Sleep Disorder” OR “Breathing Related Sleep Disorders” OR “Sleep Apnea Hypopnea” OR “Sleep Related Hypoventilation” OR “Circadian Rhythm Sleep Wake Disorders” OR Parasomnia* OR “Rapid Eye Movement Sleep” OR Sleepwalking OR Sleep-walk* OR "Sleep Terror” OR Nightmare* OR “Restless Legs Syndrome” OR Sleep-Wake* OR “Sexual Dysfunction” OR Sexually-Dysfunction* OR “Delayed Ejaculation” OR “Erectile Disorder” OR “Female Orgasmic Disorder” OR “Female Sexual Interest Arousal Disorder” OR “Genito Pelvic Pain Penetration Disorder” OR “Male Hypoactive” OR “Sexual Desire Disorder” OR “Premature Ejaculation” OR “Gender Dysphoria” OR “Disruptive, impulse Control, and Conduct Disorder” OR “Oppositional Defiant Disorder” OR “Intermittent Explosive Disorder” OR “Conduct disorder” OR Personality-disorder* OR Pyromania OR Kleptomania OR “Substance Related” OR Addictive-Disorder* OR “Alcohol Related Disorder” OR “Alcohol Related Disorders” OR “Alcohol Use Disorder” OR “Alcohol Intoxication” OR “Alcohol Withdrawal” OR “Alcohol Induced Disorder” OR “Caffeine Related Disorder” OR “Caffeine Intoxication” OR “Caffeine Withdrawal” OR “Caffeine Induced Disorder” OR “Cannabis Related Disorder” OR “Cannabis Related Disorders” OR “Cannabis Use Disorder” OR “Cannabis Use Disorders” OR “Cannabis Intoxication” OR “Cannabis Withdrawal” OR “Hallucinogen Related Disorder” OR “Hallucinogen Related Disorders” OR “Hallucinogen Intoxication” OR “Hallucinogen Persisting Perception Disorder” OR “Hallucinogen Use Disorder” OR “Hallucinogen Use Disorders” OR “Hallucinogen Induced Disorder” OR “Hallucinogen Induced Disorders” OR “Phencyclidine Use Disorder” OR “Phencyclidine Use Disorders” OR “Phencyclidine Intoxication” OR “Phencyclidine Induced Disorder” OR “Phencyclidine Related Disorder” OR “Phencyclidine Related Disorders” OR “Inhalant Related Disorder” OR “Inhalant Related Disorders” OR “Inhalant Use Disorder” OR “Inhalant Use Disorders” OR “Inhalant Intoxication” OR “Inhalant Induced Disorders” OR “Inhalant Induced Disorder” OR “Opioid Related Disorder” OR “Opioid Related Disorders” OR “Opioid Use Disorder” OR “Opioid Use Disorders” OR “Opioid Intoxication” OR “Opioid Withdrawal” OR “Opioid Induced Disorder” OR “Opioid Induced Disorders” OR “Sedative, Hypnotic or Anxiolytic” OR “Stimulant related disorders” OR “Stimulant related disorder” OR “Stimulant Use Disorder” OR “Stimulant Use Disorders” OR “Stimulant Intoxication” OR “Stimulant Withdrawal” OR “Stimulant induced disorder” OR “Stimulant induced disorders” “Tobacco related disorder” OR “Tobacco related disorders” OR “Tobacco withdrawal” OR “Tobacco induced disorder” OR “Tobacco induced disorders” OR “Substance related disorder” OR “Substance related disorders” OR “Substance intoxication” OR “Substance Withdrawal” OR “Substance induced disorder” OR “Substance induced disorders” OR Neurocognitive-Disorder* OR Deliri* OR Personality-disorder* OR “Paraphilic Disorder” OR “Voyeuristic Disorder” OR “Exhibitionistic Disorder” OR “Frotteuristic Disorder” OR “Sexual Masochism Disorder” OR “Sexual Sadism Disorder” OR “Pedophilic Disorder” OR “Paedophilic Disorder” OR “Fetishistic Disorder” OR “Transvestic Disorder” OR Mental-Disorder* OR “Medication Induced Movement Disorders” OR “Medication Induced Movement Disorder” OR “Antidepressant Discontinuation Syndrome” OR “Other Adverse Effects of Medication” OR “Other Conditions That May Be a Focus of Clinical Attention” OR “Other Problems Related to Primary Support Group” OR “Abuse and Neglect” OR “Child Physical Abuse*” OR “Child Sexual Abuse” OR “Child Neglect” OR “Child Psychological Abuse” OR “Adult Maltreatment and Neglect Problems” OR “Spouse or Partner” OR “Abuse by Nonspouse or Nonpartner” OR “Educational and Occupational Problems” OR Educational-Problem* OR Occupational-Problem* OR “Housing and Economic Problems” OR Economic-Problem* OR “Other Problems Related to the Social Environment” OR “Problems Related to Crime or Interaction With the Legal System” OR “Other Health Service Encounters for Counseling and Medical Advice” OR “Problems Related to Other Psychosocial, Personal, and Environmental Circumstances” OR “Other Circumstances of Personal History” OR “Problems Related to Access to Medical and Other Health Care” OR “Nonadherence to Medical Treatment” OR Affective-disorder* OR Mania* OR “Common mental disorder” OR “Common mental disorders” OR Internalis* OR Internaliz* OR Mood-Disorder* OR “Disruptive Mood Dysregulation Disorder” OR “Disruptive Mood Dysregulation Disorders” OR Chronic-worr* OR Panic* OR Neurosis OR Neuroses OR Neurotic OR Trauma* OR Psychotrauma* OR “Psycho trauma” OR Psychiatri* OR “Serious Emotional Disturbance” OR SED |
| Chronic Pain | “Chronic Pain” OR Pain OR “Long term pain” OR “Persistent pain” OR “Recurrent Pain” OR Headache OR “Head ache” OR “Head ache” OR Migraine OR Cephalalgi* OR Fibromyalgia OR “Stomach ache” OR “Tummy ache” OR “Abdominal ache” OR “Abdominal pain” OR “Belly ache” OR Dysmenorrh* OR Neuralgi* OR Neuropath* OR Odontalgia OR “Irritable bowel syndrome” OR IBS OR Arthrit* OR Juvenile Idiopathic Arthritis OR JIA OR Osteoarthrit* OR “Complex regional pain syndrome” OR CRPS OR “Reflex sympathetic dystrophy syndrome” OR RSD NOT Acute |
| Co-occurrence | Co occur* OR Comorbid* OR Co morbid* OR “Dual Diagnosis” OR “Multiple Disorder*” OR “Multiple Diagnosis” OR Multimorbidity |
| Function | Function* OR Academi* OR School OR Social* OR Emotion* OR “Quality of life” OR “Wellbeing” OR Disability |
