# Supplementary material for: Co‐occurring chronic pain and primary psychological disorders in adolescents: A scoping review
Source: Paediatr Neonatal Pain. 2023 May 25;5(3):57–65. doi: 10.1002/pne2.12107 (PMC10514777; doi:10.1002/pne2.12107)
Supplement: Supplementary file 3 — Appendix S3. [file PNE2-5-57-s002.docx]

| Supplementary Material 3  Articles not found |
| --- |
| Adelekan, M. L., Ndom, R. J., Ekpo, M., & Oluboka, O. (1999). Epidemiology of childhood behavioural disorders in Ilorin, Nigeria-findings from parental reports. *West African Journal of Medicine*, *18*(1), 39-48. |
| Beiter, M., Ingersoll, G., Ganser, J., & Orr, D. P. (1991). Relationships of somatic symptoms to behavioral and emotional risk in young adolescents. *The Journal of Pediatrics*, *118*(3), 473-478. |
| Sexton-Radek, K. (1995). The nature of recurrent tension headache and stress experiences. *Psychotherapy in Private Practice*, *13*(3), 63-72. |
|  |

*Note.*  Despite using the interlibrary loan resource and reaching out to authors, three articles remained elusive.
