# Supplementary material for: Co‐occurring chronic pain and primary psychological disorders in adolescents: A scoping review
Source: Paediatr Neonatal Pain. 2023 May 25;5(3):57–65. doi: 10.1002/pne2.12107 (PMC10514777; doi:10.1002/pne2.12107)
Supplement: Supplementary file 4 — Appendix S4. [file PNE2-5-57-s001.docx]

*Note. A spreadsheet with the categories as column headings was used to extract the data with one row per article.*

| Supplementary Material 4  Data Extraction Categories |  |
| --- | --- |
| Category | Detailed Description |
| Study details |  |
| Article details | The full article reference including country of where research was conducted to enable a full cultural understanding. |
| Study aims and objectives | Study aims and objectives and where possible the research question. |
| Sample |  |
| Sample size | The number of participants recruited and any individual details about separate groups (e.g., those in a co-occurring group). |
| Age range | The age range, mean and SD of all participants and those included in any groups of relevance to the review (e.g., separate reporting of those aged 11-19 with co-occurring symptoms). |
| Gender | Male, female, non-binary etc. |
| Pain condition | The pain disorders discussed in the study. |
| Psychological condition | The psychological disorders discussed in the study. |
| Inclusion criteria | The study inclusion criteria |
| Design |  |
| Longitudinal or cross-sectional | The study design for example, cross-sectional or longitudinal and details of longitudinal time points and duration. |
| Quantitative assessment methods used | The validated methods used to assess participants (e.g., RCADS, PROMIS). |
| Quantitative analytical approach | The methods used to analyse the quantitative data i.e., ANOVA, hierarchical. |
| Qualitative methods used | The qualitative methods used to collect participant data (e.g., interviews, focus groups). |
| Qualitative analytical approach | The approach used to analyse the qualitative data, (e.g., specific approaches used to analyse date, such as thematic analysis.) |
| Methodology |  |
| Assessment method used | The methods used to assess the participants for example, self-report, parent report, clinical assessment. |
| Recruitment location | The location the recruitment took place (e.g., school, clinic, hospital). |
| Study outcomes | The key findings that relate that relate to the challenges faced by youth with co-occurring psychopathology and chronic pain. |
| Author’s interpretation of findings related to functioning | The article authors interpretation of the findings that directly relate to functioning. |
| Other key findings | Any other key findings of relevance. |
